# Comparative genomic analyses reveal broad diversity in botulinum-toxin-producing *Clostridia*

**DOI:** 10.1186/s12864-016-2502-z

**Published:** 2016-03-03

**Authors:** Charles H. D. Williamson, Jason W. Sahl, Theresa J. Smith, Gary Xie, Brian T. Foley, Leonard A. Smith, Rafael A. Fernández, Miia Lindström, Hannu Korkeala, Paul Keim, Jeffrey Foster, Karen Hill

**Affiliations:** Center for Microbial Genetics and Genomics, Northern Arizona University, Flagstaff, AZ 86011 USA; Molecular and Translational Sciences Division, United States Army Medical Research Institute of Infectious Diseases, Fort Detrick, MD 21702 USA; Bioscience Division, Los Alamos National Laboratory, Los Alamos, NM 87545 USA; Theoretical Division, Los Alamos National Laboratory, Los Alamos, NM 87545 USA; Medical Countermeasures Technology, United States Army Medical Research and Material Command, United States Army Medical Research Institute of Infectious Diseases, Fort Detrick, MD 21702 USA; Área Microbiología, Departamento de Patología, Facultad de Ciencias Médicas, Universidad Nacional de Cuyo, Centro Universitario, (5500) Mendoza, Argentina; Department of Food Hygiene and Environmental Health, University of Helsinki, Helsinki, Finland; Present Address: Department of Molecular, Cellular and Biomedical Sciences, University of New Hampshire, Durham, NH 03824 USA

**Keywords:** *Clostridium botulinum*, Botulinum neurotoxin, Whole genome sequence, Comparative genomics

## Abstract

**Background:**

*Clostridium botulinum* is a diverse group of bacteria characterized by the production of botulinum neurotoxin. Botulinum neurotoxins are classified into serotypes (BoNT/A–G), which are produced by six species/Groups of *Clostridia*, but the genetic background of the bacteria remains poorly understood. The purpose of this study was to use comparative genomics to provide insights into the genetic diversity and evolutionary history of bacteria that produce the potent botulinum neurotoxin.

**Results:**

Comparative genomic analyses of over 170 *Clostridia* genomes, including our draft genome assemblies for 59 newly sequenced *Clostridia* strains from six continents and publicly available genomic data, provided in-depth insights into the diversity and distribution of BoNT-producing bacteria. These newly sequenced strains included Group I and II strains that express BoNT/A,/B,/E, or/F as well as bivalent strains. BoNT-producing *Clostridia* and closely related *Clostridia* species were delineated with a variety of methods including 16S rRNA gene, concatenated marker genes, core genome and concatenated multi-locus sequencing typing (MLST) gene phylogenies that related whole genome sequenced strains to publicly available strains and sequence types. These analyses illustrated the phylogenetic diversity in each Group and the diversity of genomic backgrounds that express the same toxin type or subtype. Comparisons of the botulinum neurotoxin genes did not identify novel toxin types or variants.

**Conclusions:**

This study represents one of the most comprehensive analyses of whole genome sequence data for Group I and II BoNT-producing strains. Read data and draft genome assemblies generated for 59 isolates will be a resource to the research community. Core genome phylogenies proved to be a powerful tool for differentiating BoNT-producing strains and can provide a framework for the study of these bacteria. Comparative genomic analyses of *Clostridia* species illustrate the diversity of botulinum-neurotoxin-producing strains and the plasticity of the genomic backgrounds in which *bont* genes are found.

**Electronic supplementary material:**

The online version of this article (doi:10.1186/s12864-016-2502-z) contains supplementary material, which is available to authorized users.

## Background

*Clostridium botulinum* encompasses diverse anaerobic, spore-forming bacteria that are defined by the production of one, two or three botulinum neurotoxins (BoNTs) [1]. The botulinum neurotoxin produces a flaccid paralysis known as botulism that affects humans, other mammals, birds and fish [2]. There are seven serotypes of botulinum neurotoxins, BoNT/A–G, produced by six known Groups of *Clostridia* [3]. Recently, sequencing of an infant botulism isolate has also identified an unusual toxin type (BoNT/H or F/A) that is produced in combination with BoNT/B [4, 5]. Group I includes BoNT/A,/B and/F-producing strains; Group II includes BoNT/B,/E and/F-producing strains; Group III includes BoNT/C and/D-producing strains; Group IV includes the BoNT/G-producing *C. argentinense* strains; Group V includes the BoNT/F-producing *C. baratii* strains; and Group VI includes the BoNT/E-producing *C. butyricum* strains [6]. BoNT/A–G are ~35–70 % different (amino acid identity) from each other and can vary within a toxin type [3]. The variants within a serotype are described by a numerical designation following the toxin such as A1, A2, A3, etc. Thus, BoNT-producing bacteria are members of at least four different bacterial species, as well as several well-defined subclades, that contain a large diversity of toxin types.

Numerous recombination events between toxins have been documented [1, 7, 8], and bivalent toxin combinations within the same strain have been identified [3]. Strains in different Groups can produce the same toxin (e.g. Group I, II and V strains produce BoNT/F). Horizontal gene transfer of the toxin gene between strains in the six Groups via toxin gene associations with transposases such as insertion sequence (IS) elements, recombinases, the acquisition of plasmids or infection by phage [9–11] is supported by incongruent topologies between the *bont* gene and 16S rRNA gene phylogenies as well as the presence of the same toxin subtype on the chromosome in some strains and on plasmids in other strains [3, 6]. Recombination among toxins and horizontal gene transfer between different species and/or Groups therefore allow for substantial variation in botulinum neurotoxins and in the genomic backgrounds in which the same toxin type or subtype are found. Thus, capturing data for both the genomic background as well as the toxin type provides valuable information about the diversity within BoNT-producing species/Groups and how this genetic and phenotypic variation is generated.

Group designations were initially established based upon biochemical and microbiological attributes of the bacteria, though the Group designations and the genetic variation of the bacteria and the toxin have been increasingly investigated using different molecular techniques [12]. The first genomic sequence of a BoNT-producing strain, *C. botulinum* ATCC 3502, was used to develop microarrays to query Group I strains [13, 14]. Later the whole genomic sequence of Eklund 17B was used to construct a DNA microarray and query Group II strains [15]. These microarray studies identified diversity in the bacteria that express the botulinum toxin and identified clades of bacteria within their collections that shared common genes. Amplified fragment-length polymorphism (AFLP) analysis has been used to examine the diversity of Group I [7] and II strains [16] and to identify the complexity of 1090 strains of neurotoxin-producing *Clostridia* primarily from California infant botulism cases [17]. Pulsed-field gel electrophoresis (PFGE) has been used to examine BoNT-producing strains [18, 19], including the determination of toxin gene cluster variation and location (plasmid or chromosome) of *bont*/B variants [20]. Multi-locus sequence typing (MLST) has been used to differentiate Group I serotype A strains [21] and Group II serotype E strains [22]. These various genetic methods provide a baseline of understanding of phylogenetic relationships among *Clostridia* species. Improved DNA sequencing technologies and bioinformatic techniques now available allow researchers to compare strains at a higher level of resolution.

Botulinum neurotoxin-producing *Clostridia* have been identified from all continents except Antarctica. These microbes are often isolated from samples associated with human botulism cases (food poisonings or intestinal or wound infections), and researchers have also isolated BoNT-producing *Clostridia* from environmental samples including soils, honey, aquatic sediments and plants [17, 23–34]. The distribution of serotypes A–G can be obtained from publications based upon characterization of strains within different culture collections, environmental sampling and reported botulism cases. Identifying BoNT-producing strains from sources around the globe aids in understanding the frequency and geographic distribution of strains containing various toxin types.

The study represents a collaborative effort among researchers at many institutions to understand the diversity within BoNT-producing *Clostridia*. The study provides genomic sequence data and draft genome assemblies for strains (predominantly belonging to *C. botulinum* Groups I and II) representing diverse serotypes and geographic regions including isolates from botulism cases and environmental sources from Argentina, Australia, Canada, Finland, France, Greenland, Japan, Mauritius, Sweden and the US. These data are useful for determining neurotoxin gene cluster characteristics of BoNT-produding strains, the genomic backgrounds containing botulinum toxin genes, and the global distribution of strains expressing different toxin types. The study demonstrates that comparative genomic techniques differentiate BoNT-producing strains (including strains expressing the same toxin type or subtype) and illustrates the diversity of BoNT-producing strains (including the diversity of strains within Groups I and II). Knowledge of the diversity and phylogenetic relationships of BoNT-producing strains provides a framework for the study of these bacteria and can inform future research regarding topics such as the development of diagnostic tools and therapeutics.

## Methods

### Genome sequencing, assembly and annotation

Strains or purified DNA were kindly provided by numerous collaborators. Whole genome sequence data were generated with the Illumina sequencing technology (GAIIx). Genomes were assembled *de novo* via an in-house pipeline [35] that included adapter trimming with Trimmomatic [36], read error correction with BayesHammer [37] and contig assembly with SPAdes v3.0.0 [38]. Redundant contigs were removed with PSI-cd-hit [39], and short contigs (<200 nt) were filtered out of assemblies. Illumina reads were mapped back to assemblies with BWA [40, 41], and single nucleotide polymorphisms were identified with GATK [42] and corrected if they passed a minimum depth and allele proportion. Assemblies were then improved with PILON [43] and IMAGE [44]. Assemblies were screened for obvious contamination by BLAST [45] searches against the NCBI non-redundant nucleotide database (contamination was removed from data for strain U21312). Relevant, published *Clostridia* species genomes were identified with the aid of the PATRIC database phylogeny viewer [46] and literature searches. Genome assemblies were downloaded from PATRIC [46] or GenBank [47] in fasta format (January 2015). The genome assembly of *Acetobacter woodii* DSM 1030 [GenBank:NC_016894] was downloaded for use as an outgroup. For inclusion in analyses, published genome assemblies needed to meet the following requirements: <800 contigs, presence of a near full-length 16S rRNA gene (>1300 nt) and presence of an *rpoB* gene. All genome assemblies (newly sequenced and published strains) were annotated with Prokka [48] and evaluated with QUAST [49]. In addition to published genome assemblies, read files for ten Group I *C. botulinum* [50] genomes were included for the core genome sequence phylogenies of Group I strains.

### 16S rRNA gene phylogeny

16S rRNA genes were parsed from Prokka output files. If multiple 16S rRNA gene sequences were present in an assembly, the sequences from that assembly were clustered (99 % identity) with USEARCH [51] and a representative sequence was chosen for that assembly. Representative sequences were aligned and masked with SSU-ALIGN [52]. Aligned and masked sequences were trimmed with mothur (filter.seqs command) [53] so the first and last position of each sequence included a base (not a gap character). A phylogeny was inferred with FastTree2 [54], an approximately-maximum-likelihood method, using the general time reversible model of nucleotide substitution and 1000 bootstrap replicates. The tree was viewed and rooted with the *A. woodii* DSM 1030 16S rRNA gene sequence in FigTree v1.4.2 [55].

### Phylogeny of concatenated marker genes

Forty marker genes identified by specI (species identification tool) [56], a software package developed to delineate microbial species, were extracted from Prokka output files. Gene sequences of sufficient length (80 % of the length of 97.5 % of the extracted sequences for each gene) were aligned with MUSCLE [57] and concatenated. The final alignment included gap characters for marker genes that could not be extracted from some genome assemblies. Alignment columns containing greater than 95 % gap characters were filtered from the alignment with QIIME v1.6.0 [58]. Phylogenies were inferred on the concatenated alignments with FastTree2 as described above. The tree was viewed and rooted with *A. woodii* DSM 1030 in FigTree.

### Single nucleotide polymorphism (SNP) detection and phylogeny

All genome assemblies were compared with the reference-independent single nucleotide polymorphism (SNP) approach, kSNP v2 [59]. kSNP was run with a kmer value of 21. SNPs identified in at least 50 % of the analyzed genomes were used to infer a phylogeny. A phylogeny was inferred on the 40,582-character matrix with FastTree2 as described above. Reference-independent SNP phylogenies were also generated for Group I and II strains. The Group I and II phylogenies were inferred upon core SNP matrices (Group I – 1,780-character matrix, Group II – 35,382-character matrix) generated by kSNP, as described above.

SNP discovery was also performed by aligning assembled genomes to a reference assembly with NUCmer [60] and identifying SNPs from these alignments with NASP [61]. Illumina reads for ten Group I strains were aligned against the reference with BWA-MEM [41] and SNPs were called with the UnifiedGenotyper method in GATK [62]. SNP calls were filtered from the final matrix if the coverage at a position was less than 10× or if the proportion of reads matching the called SNP was less than 0.9. SNPs called from duplicated regions in the reference genome (identified by self alignments with NUCmer) were filtered from the SNP matrix. Phylogenies were inferred with RAxML v8.1.1 [63] using the general time reversible model of nucleotide substitution and the gamma distribution of rate heterogeneity. Ascertainment bias correction was applied to likelihood calculations [64] within RAxML. Bootstrap replicates were conducted using the rapid bootstrapping method in RAxML [65], and the number of bootstrap replicates was determined by using the RAxML extended majority-rule consensus tree criterion [66]. The tree was viewed and rooted in FigTree. *C. botulinum* strain Kyoto-F [GenBank:CP001581] was used as the reference genome for SNP detection for Group I *C. botulinum/C. sporogenes* resulting in a 200,641-character core genome SNP matrix called from a 1,708,420-character core genome alignment (positions that passed quality filtering). The core genome phylogeny for Group I was rooted with the clade including *C. sporogenes* and *C. botulinum* serotype B strains based upon the concatenated marker genes and kSNP phylogenies of all genomes included in this study as well as reference-based SNP phylogenies including *C. tetani* strains as an outgroup (data not shown). *C. botulinum* strain Eklund 17B [GenBank:CP001056, CP001057] was used as the reference genome for SNP detection for Group II *C. botulinum* resulting in a 197,688-character SNP matrix called from a 2,609,405-character core genome alignment. The core genome phylogeny for Group II was rooted with the clade containing strains Eklund 202F, KAPB-3, Eklund 17B and CDC 66177 based upon the concatenated marker genes and kSNP phylogenies of all the genomes included in this study as well as reference-based SNP phylogenies including *C. saccharobutylicum* DSM 13864 [GenBank:CP006721] as an outgroup (data not shown). Reference-based SNP detection and core genome phylogenies with alternate reference genomes were also generated: Group I – *C. sporogenes* ATCC 15579 [GenBank:ABKW00000000], Group II – *C. botulinum* strain Alaska E43 [GenBank:CP001078].

### Analysis of phylogenies

The consistency index and retention index for core genome phylogenies was computed with the R [67] package phangorn [68]. Compare2Trees [69] was used to compare tree topologies for Groups I and II core genome phylogenies and for 16S rRNA gene, concatenated marker genes and kSNP phylogenies. The overall topological score is reported as a measure of tree topology similarity.

### SNP and homoplasy density in *C. botulinum* Groups I and II

SNP density and homoplasy density ratio were computed using the SNP matrices and core genome phylogenies produced by NASP and RAxML (see above) to provide insight into recombination within Groups I and II. SNP density was determined by counting the number of parsimony informative SNPs present in 1 kb non-overlapping segments of the core genome for each Group. The homoplasy density ratio was computed by dividing the number of parsimony informative SNPs with a retention index below 0.5 (calculated with PAUP* 4.0 beta [70]) by the total number of parsimony informative SNPs in 1 kb segments of the core genome for each Group. The SNP density and homoplasy density ratio values across the reference genomes were plotted with Circos [71]. Histograms of the homoplasy density ratio values of 1 kb segments of the core genome (only 1 kb segments with at least ten parsimony informative SNPs are included in the histogram) are presented for Groups I and II.

### Pairwise genomic comparisons – average nucleotide identity

The average nucleotide identity between pairs of genome assemblies (analysis included chromosomal and extra-chromosomal sequences) was computed with JSpecies [72] using the MUMmer calculation (ANIm) and default settings. A histogram of ANIm values of inter- and intra-Group comparisons of *C. botulinum* Groups I, II, III and VI as well as *C. perfringens* and *C. tetani* was created with matplotlib [73]. These Groups were chosen for comparison because they contain strains that produce botulinum neurotoxins or the tetanus toxin and/or have multiple sequenced genomes within the Group/species.

### Gene content analyses with LS-BSR

Genome assemblies were processed with the large-scale BLAST score ratio pipeline (LS-BSR) [74] using the BLAT [75] alignment option and default parameters to assess the gene content of Group I and II strains. Group I and II genomes were clustered based on BSR values using an average linkage algorithm implemented in the MultiExperiment Viewer (MeV) [76]. Dendrograms produced by MeV were viewed and rooted in Figtree. Additionally, cold shock protein encoding genes were screened against Group II genomes using the LS-BSR approach. Cold shock protein encoding genes were downloaded from PATRIC [46] for *C. botulinum* strains ATCC 3502 (PATRIC IDs fig|413999.7.peg.282, fig|413999.7.peg.1366, fig|413999.7.peg.1745), Eklund 17B (fig|508765.6.peg.1446) and Eklund (fig|445337.5.peg.496, fig|445337.5.peg.1216) as well as *C. beijerinckii* strain NCIMB 8052 (fig|290402.41.peg.2890, fig|290402.41.peg.3037), and *C. butyricum* strains 5521 (fig|447214.4.peg.3792) and BL5262 (fig|632245.3.peg.2731).

### Phylogeny of concatenated multi-locus sequence typing (MLST) genes

Genes for multilocus-sequence typing (MLST) were selected from previous MSLT studies. The MLST profile for Group I strains (*aceK*, *aroE*, *hsp*, *mdh*, *oppB*, *recA* and *rpoB*) was selected from Jacobson and colleagues [21]. The MLST profile for Group II strains (16S rRNA gene, *atpD*, *guaA*, *gyrB*, *ilvD*, *lepA*, *oppB*, *pta*, *pyc*, *recA*, *rpoB*, *trpB* and *tuf*) was adapted from MacDonald and colleagues [22], though 23S rRNA gene sequences were not included in the analysis. These gene sequences were downloaded from PubMLST *C. botulinum* database [77, 78] or GenBank. MLST genes were extracted from genome assemblies with BLAST searches. Gene sequences were aligned with MUSCLE and concatenated for phylogenetic reconstruction. Phylogenies were inferred with FastTree2 as described above (see 16S rRNA gene phylogeny methods). Two genome assemblies included in the Group I analyses were missing one gene from the MLST profile – *C. botulinum* CDC 54091 had no *recA* gene and *C. botulinum* Af84 had no *mdh* gene. Gap characters were inserted into the gene alignments for these two genomes.

### *bont* gene cluster analyses

Botulinum neurotoxin gene sequences were extracted from Prokka output files. Previously published *bont* gene and tetanus toxin gene sequences downloaded from GenBank were also included in the analyses. Sequences longer than 3500 nucleotides were aligned with MUSCLE and trimmed with Mothur (filter.seqs command) so the first and last position of each sequence included a base (not a gap character). A phylogeny was constructed with Fasttree2 as described above (see 16S rRNA gene phylogeny methods). The tree was viewed and rooted with the tetanus toxin gene clade in FigTree. Annotated genome assemblies were investigated to determine the putative *bont* gene cluster type (*ha*+ or *orfX*+) and location within the genome. Newly sequenced genomes were aligned to previously published genome assemblies with progressiveMauve [79] to aid in understanding putative *bont* gene cluster locations. The presence of a plasmid-specific marker gene (*PL-6*) in all genome assemblies was determined with BLAST searches (hits above 80 % identity) of assembled genomes against a putative DNA primase gene [GenBank:CP000940.1, locus CLD_A0039] [80].

## Results

**Fig. 1:**
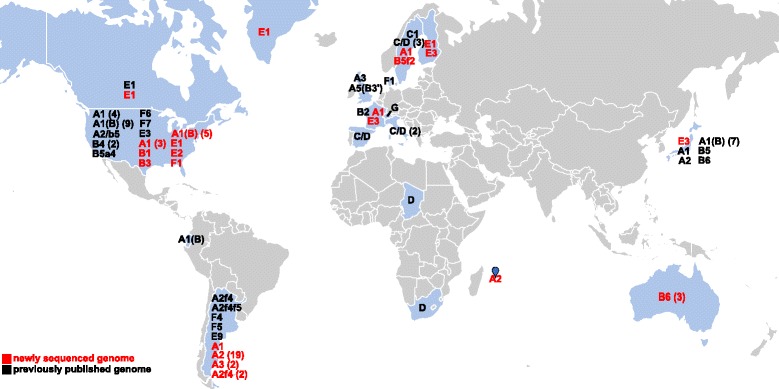
World map indicating the provenance of whole genome sequenced BoNT-producing strains analyzed in this study. Strains in red were sequenced as part of this study. Strains in black are previously published strains. Countries from which sequenced strains originated are *colored blue*. Numbers in parentheses indicate how many strains of each subtype are included in this study. Blank map downloaded from amcharts.com

A total of 59 new draft genome assemblies were generated for strains isolated from six different continents (Table 1 and (Additional file 1: Table S1)). The strains were previously isolated from botulism cases and environmental samples and include 32 *bont*/A, six *bont*/B, nine *bont*/E, one *bont*/F, five *bont*/A1(B), two bivalent *bont*/A2f4, one bivalent *bont*/Bf and three strains that did not contain botulinum neurotoxin genes in the draft genome assemblies (two strains within Group I and one strain not within the six BoNT-producing Groups). Information regarding previously published genomes included in this study is presented (Additional file 2: Table S2). Fig. 1 illustrates the countries of origin of BoNT-producing strains sequenced and/or analyzed in this study and demonstrates the small number of whole genome sequences available for strains originating from Asia and Africa. This study is one of the most comprehensive comparative genomic analyses of *C. botulinum* and closely-related strains performed to date.

**Table 1:** Information regarding newly sequenced strains

Genome	Accession #	Group	BoNT	Strain/alternate ID	BoNT cluster type^a^	BoNT cluster location^a^	Genomic site^a^	Year	Origin	Location
C. botulinum 20386	LFRD00000000	I	A1	VPI 7124	ha+	chr	oppA/BrnQ		soil	USA:Virginia
C. botulinum 20389	LFOO00000000	I	A1	ATCC 449	ha+	chr	oppA/BrnQ			
C. botulinum 20412	LFOT00000000	I	A1	KF Meyer 126	ha+	chr	oppA/BrnQ	1921	spinach (FB)	USA:Indiana
C. botulinum 20414	LFOU00000000	I	A1	Prevot 910	ha+	chr	oppA/BrnQ	1953	bovine botulism	France
C. botulinum 20424	LFOV00000000	I	A1	Prevot Dewping	ha+	chr	oppA/BrnQ			
C. botulinum 20427	LFOW00000000	I	A1	Prevot 697B	ha+	chr	oppA/BrnQ	1952	cat gut	Sweden
C. botulinum 20503	LFOY00000000	I	A1	McClung 844	ha+	chr	oppA/BrnQ	<1930		
C. botulinum 20504	LFOZ00000000	I	A1	KF Meyer 33	ha+	chr	oppA/BrnQ	1920	ripe olives	USA:Tennessee
C. botulinum AM1295	LFPI00000000	I	A1		ha+	chr	oppA/BrnQ		suspected reference strain	
C. botulinum U21312	LFQF00000000	I	A1	SU0729	orfX+	chr	arsC	1987	soil	Argentina
C. botulinum 10148	LFOK00000000	I	A1(B)	CDC 1744	A1-orfX+, (B)-ha+	chr	A1-arsC, (B)-oppB/BrnQ	1977	IB	USA:Pennsylvania
C. botulinum 20391	LFOP00000000	I	A1(B)	Hall 183	A1-orfX+, (B)-ha+	chr	A1-arsC, (B)-oppB/BrnQ	1922	corn (FB)	USA:Colorado
C. botulinum 20396	LFOQ00000000	I	A1(B)	Hall 4834	A1-orfX+, (B)-ha+	chr	A1-arsC, (B)-oppB/BrnQ	1931	spinach (FB)	USA:Nebraska
C. botulinum 20397	LFOR00000000	I	A1(B)	Hall 8388A	A1-orfX+, (B)-ha+	chr	A1-arsC, (B)-oppB/BrnQ	1935	chili pepper (FB)	USA:New Mexico
C. botulinum 20398	LFOS00000000	I	A1(B)	Hall 8857Ab	A1-orfX+, (B)-ha+	chr	A1-arsC, (B)-oppB/BrnQ	1935	corn (FB)	USA:Nebraska
C. botulinum Mauritius	LFPL00000000	I	A2	Mauritius	orfX+	chr	arsC		fish (FB)	Mauritius
C. botulinum U21063	LFPN00000000	I	A2	SU1937	orfX+	chr	arsC	2009	soil	Argentina
C. botulinum U21067	LFPQ00000000	I	A2	SU1274	orfX+	chr	arsC	1997	soil	Argentina
C. botulinum U21068	LFPR00000000	I	A2	SU1917	orfX+	chr	arsC	2009	soil	Argentina
C. botulinum U21069	LFPS00000000	I	A2	SU1887	orfX+	chr	arsC	2007	soil	Argentina
C. botulinum U21070	LFPT00000000	I	A2	SU1275	orfX+	chr	arsC	1997	soil	Argentina
C. botulinum U21075	LFPU00000000	I	A2	SU1934	orfX+	chr	arsC	2010	soil	Argentina
C. botulinum U21077	LFQY00000000	I	A2	SU1259	orfX+	chr	arsC	1996	soil	Argentina
C. botulinum U21078	LFQZ00000000	I	A2	SU1891	orfX+	chr	arsC	2007	soil	Argentina
C. botulinum U21082	LFPW00000000	I	A2	SU1054	orfX+	chr	arsC	1998	soil	Argentina
C. botulinum U21084	LFPX00000000	I	A2	SU1072	orfX+	chr	arsC	1998	soil	Argentina
C. botulinum U21086	LFPY00000000	I	A2	SU1064	orfX+	chr	arsC	1998	soil	Argentina
C. botulinum U21088	LFPZ00000000	I	A2	SU1074	orfX+	chr	arsC	1998	soil	Argentina
C. botulinum U21089	LFQA00000000	I	A2	SU1112	orfX+	chr	arsC	1995	soil	Argentina
C. botulinum U21306	LFRC00000000	I	A2	SU0801	orfX+	chr	arsC	2010	soil	Argentina
C. botulinum U21307	LFQB00000000	I	A2	SU0998	orfX+	chr	arsC	1994	soil	Argentina
C. botulinum U21309	LFQC00000000	I	A2	SU0807	orfX+	chr	arsC	1987	soil	Argentina
C. botulinum U21310	LFQD00000000	I	A2	SU0994	orfX+	chr	arsC	1994	soil	Argentina
C. botulinum U21311	LFQE00000000	I	A2	SU0634	orfX+	chr	arsC	1980	soil	Argentina
C. botulinum U21729	LFQG00000000	I	A2	SU0635W	orfX+	chr	arsC		soil	Argentina
C. botulinum U21064	LFPO00000000	I	A2f4	SU1306	A2-orfX+, F4-orfX+	chr	A2-arsC, F4-PulE	1998	soil	Argentina
C. botulinum U21076	LFPV00000000	I	A2f4	SU1304	A2-orfX+, F4-orfX+	chr	A2-arsC, F4-PulE	1998	soil	Argentina
C. botulinum U21087	LFRA00000000	I	A3	SU1169	orfX+			1998	soil	Argentina
C. botulinum U21181	LFRB00000000	I	A3	SU0945	orfX+			1992	soil	Argentina
C. botulinum 20497	LFOX00000000	I	B1	Hall 80	ha+	chr	oppA/BrnQ	1918	beans (FB)	USA:Illinois
C. botulinum 10159	LFOL00000000	I	B2	ATCC 17843 (B5)	ha+	chr	oppA/brnQ			
C. botulinum 20506	LFQV00000000	I	B3	CDC 795	ha+	chr	oppA/BrnQ			USA:Michigan
C. botulinum 10258	LFON00000000	I	B5f2	An436	B5-ha+, F2-orfX+	plasmid			IB	Sweden
C. botulinum AM1195	LFPH00000000	I	B6	AM1195	ha+	plasmid		1987	IB	Australia
C. botulinum AM370	LFPJ00000000	I	B6	AM370	ha+	plasmid		1979	salted fish	Australia
C. botulinum AM553	LFPK00000000	I	B6	AM553	ha+	plasmid		1981		Australia
C. botulinum 20552	LFPF00000000	I	F1	Walls 8G	orfX+	chr	arsC	1968	crabs (ENV)	USA:Virginia
C. sp. U21066	LFPP00000000	I	NT	SU1575NT				1999	soil - colony variant of SU1575	Argentina
C. sporogenes U20719	LFPM00000000	I	NT	ATCC 19404						
C. botulinum 20536	LFPA00000000	II	E1	CDC KA-95B	orfX+	chr	rarA			
C. botulinum 20541	LFPB00000000	II	E1	L-572	orfX+	chr	rarA		forest soil	USA:Washington
C. botulinum 20547	LFPD00000000	II	E1	Prevot Ped 1	orfX+	chr	rarA		sea mud	Greenland
C. botulinum 20675	LFPG00000000	II	E1	ATCC 9564	orfX+	chr	rarA	1961	smoked salmon	Canada
C. botulinum K15	LFQW00000000	II	E1	K15	orfX+	chr	rarA	1995	trout	Finland
C. botulinum 20544	LFPC00000000	II	E2	CDC 5247	orfX+	chr	rarA			USA:Alaska
C. botulinum 10178	LFOM00000000	II	E3	211	orfX+	chr	rarA		lake sediment	Japan
C. botulinum 20549	LFPE00000000	II	E3	Prevot R81-3A	orfX+	chr	rarA			France
C. botulinum K3	LFQX00000000	II	E3	K3	orfX+	chr	rarA	1995	trout	Finland
C. sp. U20725	LFRG00000000		NT	ATCC 25772						

*FB* foodborne isolate, *IB* infant botulism case, *ENV* environmental isolate

^a^putative information based upon draft genome assemblies

### Delineation of *Clostridia* species/Groups

**Fig. 2:**
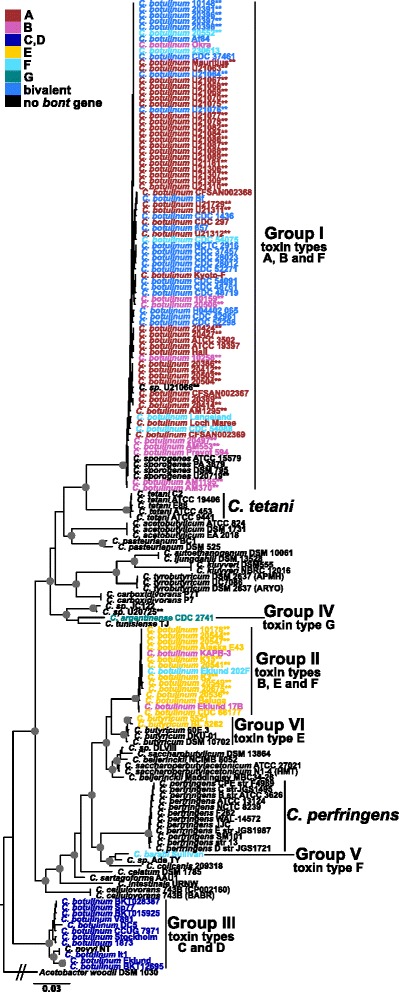
16S rRNA gene phylogeny of *Clostridia* strains. A phylogeny inferred with FastTree2 [54] on near-full-length 16S rRNA gene sequences extracted from genome assemblies and aligned and masked with ssu-align [52]. The tree is rooted with *Acetobacter woodii* DSM 1030 [GenBank:NC_016894]. Strains are color-coded by botulinum neurotoxin serotype. Stars indicate newly sequenced (this study) strains. Groups I–VI, *Clostridium perfringens* and *C. tetani* clades are labeled. *Gray circles* indicate bootstrap values over 90 %

The phylogenetic relationships and diversity of BoNT-producing strains were evaluated with phylogenies of the 16S rRNA gene, concatenated marker genes, and SNPs and with average nucleotide identity. A phylogeny of 16S rRNA gene sequences extracted from each genome assembly indicates that the newly sequenced strains (except for non-BoNT-producing strain U20725 that groups near the Group IV strain in Fig. 2) are closely related to strains within *C. botulinum* Groups I and II (Fig. 2). Group I includes serotypes A, B and F as well as bivalent and non-toxic strains. Group II includes serotypes B, E and F. BoNT-producing strains are color-coded by serotype in Fig. 2, which illustrates evidence of horizontal gene transfer and that non-BoNT-producing strains are closely related to BoNT-producing strains (e.g. *C. sporogenes* in Group I and *C. novyi* in Group III).

**Fig. 3:**
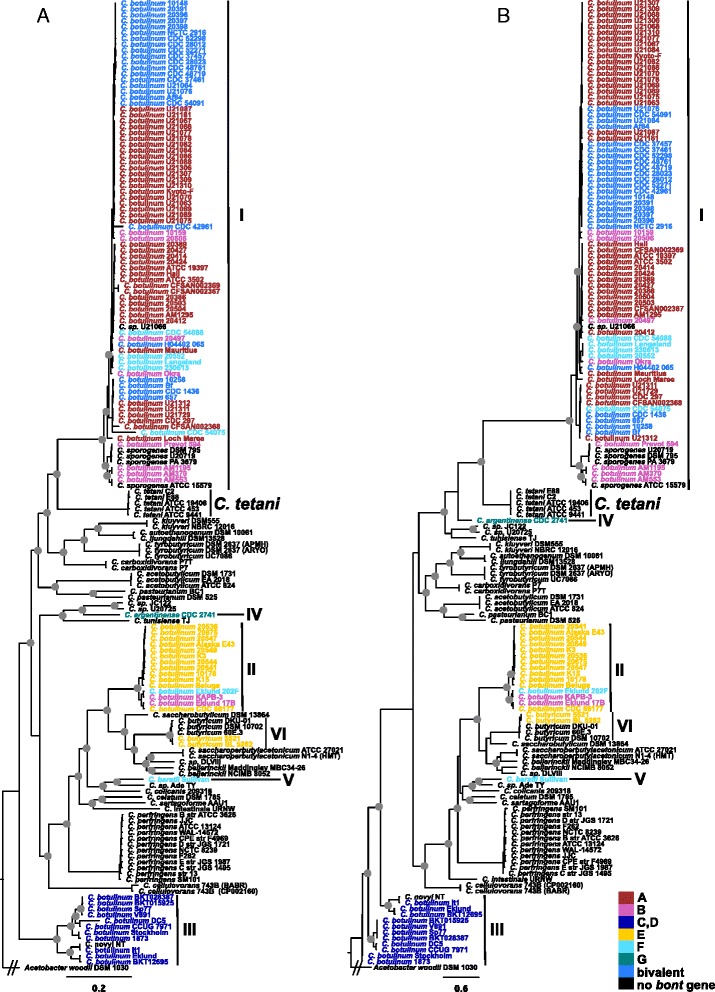
Phylogenies of *Clostridia* strains inferred on (**a**) concatenated marker genes and (**b**) a SNP matrix. Phylogenies inferred with FastTree2 [54] on (**a**) 40 concatenated marker genes for the delineation of species (specI) [56] aligned with MUSCLE [57] and (**b**) a SNP-matrix including SNPs identified in at least 50 % of the analyzed strains produced by kSNP v2 [59]. The trees are rooted with *Acetobacter woodii* DSM 1030 [GenBank:NC_016894]. Strains are color-coded by botulinum neurotoxin serotype. Groups I-VI, *Clostridium perfringens* and *C. tetani* clades are labeled. *Gray circles* indicate bootstrap values over 90 %

The deeper relationships of BoNT-producing *Clostridia* strains were also evaluated with phylogenies inferred on a concatenation of 40 marker genes identified by Mende and colleagues [56] for delineation of species (i.e. universal MLST-like approach for identifying microbial species) and a SNP-matrix produced by kSNP (Fig. 3). The 16S rRNA gene, concatenated marker genes and kSNP phylogenies show similar overall topologies (overall topological scores computed by Compare2Trees range from 66 to 78 %). All phylogenies indicate that Group III *C. botulinum* strains are an outgroup to all other BoNT-producing strains and that a close relationship exists between *C. tetani* strains and *C. botulinum* Group I strains. The concatenated marker genes and kSNP phylogenies provide greater resolution than the 16S rRNA gene phylogeny and indicate that multiple clades are present in *C. botulinum* Groups I, II and III, which is consistent with previous findings [11, 21, 22, 50, 81–84]. Among the biggest differences between the trees is the inclusion of the Group IV *C. argentinense* strain in a clade with the Group I strains in the 16S rRNA gene and kSNP trees, while the same Group IV *C. argentinense* strain falls into a clade with Groups II, V, VI in the concatenated marker genes phylogeny.

**Fig. 4:**
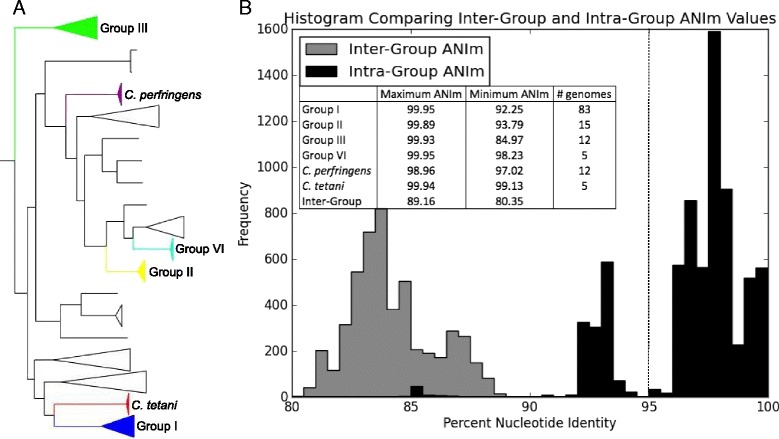
Pairwise genomic comparisons with average nucleotide identity. **a** A dendrogram highlighting clades of *Clostridia* compared by the average nucleotide identity (MUMmer calculation) method (ANIm) with Jspecies [72] and included in the histogram presented in (**b**). **b** A histogram of inter-Group and intra-Group ANIm values. The vertical dotted line at 95 % nucleotide identity indicates the cutoff for species delineation suggested by Richter and Rosello-Mora [72]. The inset table displays maximum and minimum percent nucleotide identity within each analyzed clade and between all analyzed clades

Average nucleotide identity (MUMmer method - ANIm), a method that can be applied to delineate species [72], was calculated to determine diversity at the genomic level within and between BoNT-producing Groups (Fig. 4 and (Additional file 3: Table S3)). Richter and Rossello-Mora [72] suggested that ANIm values above 95–96 % may be applied to define species, though the authors noted some exceptions, and ANIm values between 93 and 96 % may fall into an intermediate zone of species classification [85]. Minimum ANIm values in Groups I, II and III fall below the threshold of 95–96 %, and minimum ANIm values for Group I and Group III fall below 93 %, which is indicative of the relatively high diversity within these Groups [11, 83, 84]. For comparison, the minimum ANIm values for strains within *C. perfingens* and *C. tetani* (97.02 and 99.13, respectively) are well above the suggested species cutoff value, and looking outside of the *Clostridia*, *Escherichia coli* O157:H7 str. EC869 and *E. fergusonii* ATCC 35469 share 92.56 ANIm (data not shown). Multiple clades are present in *C. botulinum* Groups I and II in the concatenated marker genes and kSNP phylogenies (Fig. 3) as well as in core genome phylogenies (discussed below). While minimum ANIm values fall below the species delineation value when all genomes in either Group I or Group II are considered, ANIm values are above 95 % within each of the two distinct clades present in Group I, and ANIm values are above 97 % within each of the two distinct clades present in Group II. By this measure, both Groups I and II could be considered to encompass multiple species, subspecies or genomovars (distinct groups on the genomic level but similar phenotypically) [86]. Regardless of the assigned nomenclature, the genomic diversity within the Groups is evident.

### Phylogeny of Group I *C. botulinum/C. sporogenes*

**Fig. 5:**
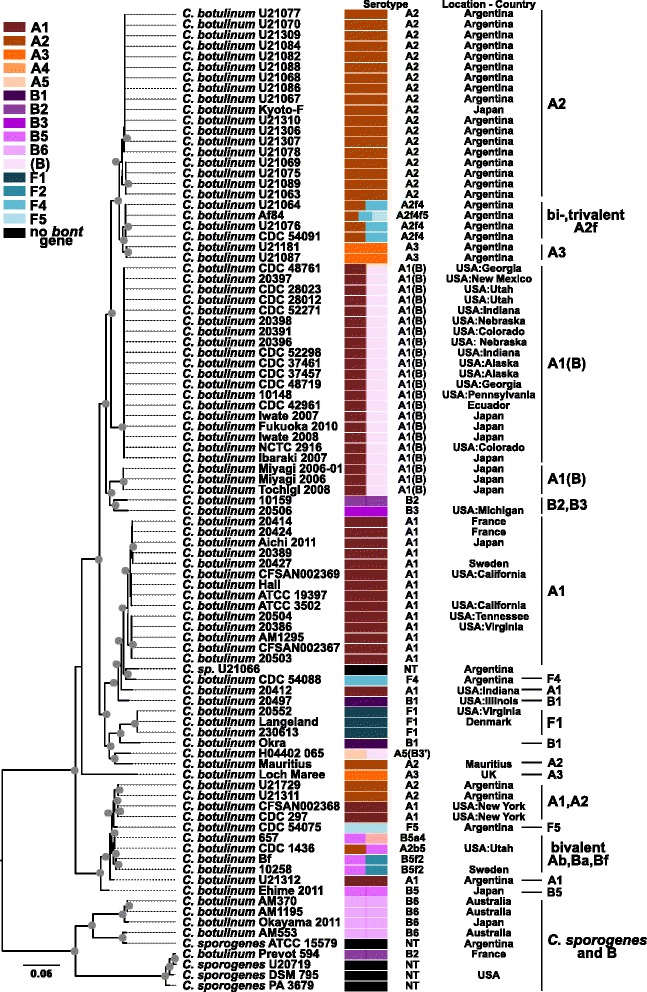
Group I core genome phylogeny. Core genome phylogeny of *C. botulinum* Group I inferred with RAxML v8.1.1 [63] using the ASC_GTRGAMMA model on an alignment of 200,641 core genome SNPs produced with NASP [61] using *C. botulinum* strain Kyoto-F [GenBank:CP001581] as a reference genome. The consistency index is 0.57, and the retention index is 0.91. *Gray circles* indicate bootstrap values over 95 %. The phylogeny was rooted with the clade that includes *Clostridium sporogenes* and *C. botulinum* B serotypes (*bottom* of Figure) using FigTree [55]. Strains are color-coded by botulinum neurotoxin serotype. Additional information regarding *bont* gene cluster characteristics is included in Table 1

Many of the newly sequenced strains belong to the Group I *C. botulinum/C. sporogenes*. To provide a high-resolution investigation of the relationships of 93 Group I strains, a maximum likelihood phylogeny was estimated from an alignment of ~200,000 core genome SNPs (Fig. 5). Core genome phylogenies generated with an alternative reference genome as well as kSNP are also presented (Additional file 4: Figure S1). Group I includes diverse BoNT/A,/B and/F-producing strains as well as non-BoNT-producing strains that fall into multiple clades, which is consistent with previous studies [1, 50, 82, 84]. When considering ANIm values, strains within the *C. sporogenes*-BoNT/B-producing outgroup (bottom of Fig. 5) share ANIm values above 95 %. The strains in the remainder of the tree also share ANIm values above 95 %. However, when comparing all Group I strains ANIm values fall below 95 % (minimum of ~92.2 %) (as mentioned above), indicating the high genomic diversity present in Group I. The core genome phylogeny provides a framework for investigating Group I strains including the variation in genomic backgrounds expressing the same toxin type or subtype.

BoNT/A-producing strains (subtypes A1, A2 and A3) belong to multiple clades of the Group I core genome phylogeny presented in Fig. 5. Subtype A1 strains show considerable genomic diversity. The newly sequenced *orfX+ bont*/A1 strain U21312 from Argentina belongs to a clade that includes the *orfX*+ *bont*/A1 strain CDC 297 (two assemblies for this strain from New York, labeled CDC 297 and CFSAN002368, were included to compare results when analyzing the same strain sequenced and assembled by different groups and methods – the strains group together in the phylogeny) but is less closely related to strain CDC 297 than other *bont*/A2 and bivalent strains. Two clades of *bont*/A1(B) strains (*orfX*+ *bont* A1) are found in the Group I phylogeny. Five *bont*/A1(B) strains (10148, 20397, 20398, 20396 and 2039) from the United States are closely related to other *bont*/A1(B) strains from Ecuador, Japan and the US while three recently published *bont*/A1(B) strains isolated from infant botulism cases in Japan [50] fall into a distinct clade. The *ha+ bont*/A1 strains included in this study belong to one clade that also includes a *bont*/B1 strain, a *bont*/F4 strain and a strain that does not contain a botulinum neurotoxin gene (strain U21066 is a colony variant of a *bont*/A strain). Three *ha+ bont*/A1 subclades include previously published genomes, but strain 20412 isolated from spinach associated with a food botulism case in the US (Indiana) is less closely related to these previously sequenced *ha+ bont*/A1 strains.

Argentinian subtype *bont*/A2 strains analyzed in this study were all isolated from soils. Many of the newly sequenced *bont*/A2 strains are closely related to the *bont*/A2 strain *C. botulinum* Kyoto-F (Fig. 5). However, the newly sequenced *bont*/A2 Argentinian strains U21069, U21089, U21063 and U21075 form a distinct but closely related clade to the *bont*/A2 group that includes the Kyoto-F strain. Additionally, two newly sequenced Argentinian *bont*/A2 strains (U21311 and U21729) are distantly related to the previously mentioned *bont*/A2 strains, but appear to be more closely related to *orfX+ bont*/A1 and bivalent strains. One newly sequenced *bont*/A2 strain from the Republic of Mauritius, an island country off the eastern coast of Africa, is not closely related to any of the Argentinian *bont*/A2 strains or the Kyoto-F strain. The diversity of the genomic backgrounds in which *bont*/A2 genes are found is evident from this analysis.

Prior to this study, only one genome assembly for a strain producing BoNT/A3, *C. botulinum* strain Loch Maree isolated in Scotland [87, 88], had been published. The Loch Maree strain is not closely related to any other whole genome sequenced strain (Fig. 5). Two Argentinian *bont*/A3 strains (U21087 and U21181) sequenced as part of this study are distantly related to the Loch Maree strain and are most closely related to bivalent *bont*/Af strains from Argentina.

BoNT/B-producing strains are also found in multiple clades in the Group I phylogeny. The BoNT/B1-producing strain 20497 is not closely related to the published whole-genome-sequenced BoNT/B1-producing strain, *C. botulinum* Okra, but instead falls within a clade that includes *ha+ bont*/A1 strains. Newly sequenced BoNT/B2-producing strain 10159 and BoNT/B3-producing strain 20506 are not closely related to other BoNT/B-producing strains. The distantly related clade at the bottom of the Group I core genome phylogeny includes *C. sporogenes* strains and *C. botulinum* strains that produce BoNT/B2 and/B6 subtypes. The newly sequenced strains include three BoNT/B6-producing strains from Australia that are closely related to the Japanese BoNT/B6-producing strain Okayama 2011 (and Japanese *bont*/B6 strain Osaka05 not included in this study) [50]. Interestingly, the acquisition or loss of the ability to produce BoNT/B within the *C. sporogenes-bont*/B clade appears to be plasmid-mediated, which is consistent with findings of Weigand and colleagues [84]; although, evaluation of additional strains would be needed to confirm this observation.

The *bont*/F1 strain 20552 (from the United States) is closely related to previously published *bont*/F1 strains F230613 and Langeland (from Denmark). Strain 20552 was isolated from an environmental source (crabs) while the Langeland strain was isolated from duck liver paste associated with a food botulism case. The *bont*/F4 strain CDC 54088 and *bont*/F5 strain CDC 54075 (both from Argentina) are found in different clades and are not closely related to the serotype F1 strains.

Bivalent strains are found within two clades in the Group I phylogeny. A clade of closely related bivalent strains is nested within the clade just above the basal clade at the bottom of Fig. 5. This bivalent clade includes two strains that produce both BoNT/A and/B (strains CDC 657 and CDC 1436) as well as the BoNT/B5f2-producing strains 10258 (from Sweden) and Bf. The second bivalent clade includes *bont*/A2f4 strains U21064 and U21076 from Argentina that are closely related to previously published bivalent *bont*/A2f4 and trivalent *bont*/A2f4f5 strains also from Argentina.

The core genome phylogeny illustrates the diversity of whole genome sequenced Group I strains. MLST is a comparative method available to many laboratories that does not require whole genome sequencing. To allow for the comparison of strains included in this study with published MLST data, a phylogeny of concatenated MLST genes including 83 sequence types (ST) available through the PubMLST *C. botulinum* database [77, 78] is included (Additional file 5: Figure S2). Similar to the core genome phylogeny (Fig. 5), the concatenated MLST gene phylogeny separates the Group I strains into two main clades. Sequence types associated with BoNT-producing and non-BoNT-producing strains fall into both main clades. *C. sporogenes* and BoNT/B-producing strains and two sequence types associated with serotype A strains (ST5 and ST17) fall into the clade corresponding to the *C. sporogenes* and *bont*/B strain clade in the core genome phylogeny (Fig. 5). Genome assemblies were not available for the two serotype A strains in this clade that might assist in further understanding the relationships of these strains.

### Phylogeny of Group II *C. botulinum*

**Fig. 6:**
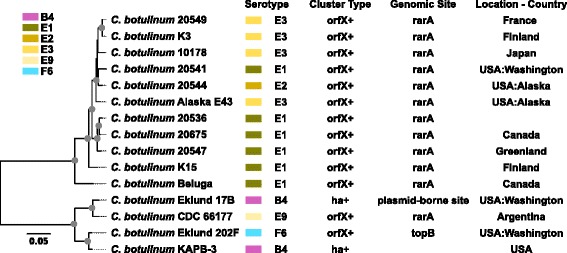
Group II core genome phylogeny. Core genome phylogeny of *C. botulinum* Group II inferred with RAxML v8.1.1 [63] using the ASC_GTRGAMMA model on an alignment of 197,688 core genome SNPs produced with NASP [61] using *C. botulinum* Eklund 17B [GenBank:CP001056, CP001057] as a reference genome. The consistency index is 0.81, and the retention index is 0.90. *Gray circles* indicate bootstrap values over 95 %. The phylogeny was rooted with the clade that includes strains Eklund 202F, KAPB-3, Eklund 17B and CDC 66177 using FigTree [55]. Strains are color-coded by botulinum neurotoxin serotype

Newly sequenced strains also included BoNT/E-producing isolates belonging to the Group II *C. botulinum*. The relationships and diversity of 15 Group II strains were investigated with a maximum likelihood phylogeny inferred from an alignment of ~200,000 core genome SNPs (Fig. 6). Phylogenies generated with an alternate reference genome as well as kSNP are also presented (Additional file 6: Figure S3). Two distinct clades are apparent in the Group II phylogeny. ANIm values are above 97 % within each major clade but fall below the species-delineating threshold of 95 % (minimum of ~93.8 %) when all Group II strains are considered, which illustrates the genomic diversity within the Group. The two major clades include a clade of only BoNT/E-producing strains and a clade of BoNT/B,/E and/F-producing strains. The newly sequenced serotype E strains fall into a clade comprised entirely of BoNT/E-producing strains representing Canada, Finland, France, Greenland, Japan and the US. The second major clade includes BoNT/B4,/E9 and/F6-producing strains. The BoNT/E9-producing strain CDC 66177 from Argentina falls into the clade with BoNT/B and/F-producing strains from the US, which is consistent with microarray hybridization profile analysis [83] and SNP analysis [89].

To allow for comparison of strains included in this study with published MLST data for other strains, a phylogeny of concatenated MLST genes including 41 *C. botulinum* serotype E strains presented in MacDonald et al. [22] is included (Additional file 7: Figure S4). Similar to the core genome phylogeny, the MLST phylogeny separates the Group II strains into two main clades: one that includes only *bont*/E strains and one that includes *bont*/B and/F strains as well as the *bont*/E9 CDC6617 strain.

### SNP and homoplasy density in *C. botulinum* Groups I and II

**Fig. 7:**
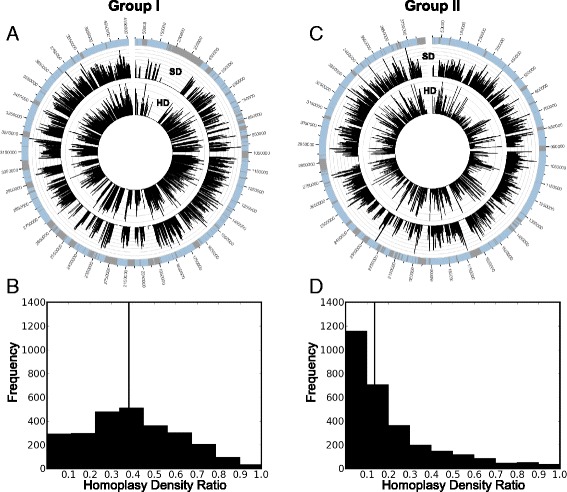
Single nucleotide polymorphism density and homoplasy density in Group I and Group II. **a** A map of SNP density and homoplasy density in Group I genomes. The *outer ring* displays the reference genome (*C. botulinum* strain Kyoto-F). *Blue shaded* regions indicate the core genome of Group I (only the core genome was considered for this analysis). The *middle ring* displays SNP density (SD), which is defined here as the number of parsimony informative SNPs found in 1 kb segments of the core genome. The *inner ring* displays the homoplasy density (HD), which is defined as the proportion of parsimony informative SNPs with a retention index below 0.5 for 1 kb segments of the core genome. **b** Histogram displaying the counts of 1 kb genome segments with homoplasy densities between 0 and 1 for Group I genomes. The *vertical line* indicates the median homoplasy density value. **c** A map of SNP and homoplasy density in Group II genomes. The *outer ring* displays information about the reference genome (*C. botulinum* strain Eklund 17B). *Blue shaded* regions indicate the core genome of Group II. The *middle ring* displays the SNP density and the inner ring displays the homoplasy density. **d** Histogram displaying the counts of 1 kb genome segments with homoplasy densities between 0 and 1 for Group II genomes. The *vertical line* indicates the median homoplasy density value. The figures suggest that SNPs and recombination are spread throughout the core genomes of each Group

Recombination is a common evolutionary process [90] that has been detected among *C. botulinum* strains [21] and may impact accurate phylogenetic inference. Regions of the genome with high proportions of homoplasious SNPs (shared SNP alleles found in different lineages of a phylogeny not inherited from a common ancestor) may indicate regions of recombination [91]. For Groups I and II, parsimony informative SNPs (sites at which at least two SNP alleles are present in at least two genomes) and putative recombination, indicated by regions of the genome with high proportions of homoplasious SNPs, appear to be distributed throughout the core genome (Fig. 7a and c). A comparison of the histograms of homoplasy density ratio values for 1 kb segments of the core genomes for Groups I and II (Fig. 7b and d) suggests that recombination is more prevalent in Group I strains as compared to Group II strains. It should be noted that fewer Group II strains were evaluated and the underlying reasons for the apparent difference in the prevalence of recombination in each Group is unknown. Additionally, the approach to detect recombination used here does not address potential recombination (or lack thereof) between or within specific clades of the Group I or Group II phylogenies. Additional analyses could provide more information about recombined regions in BoNT-producing clades (e.g. are certain types of genes more impacted by recombination events).

### Gene content of *C. botulinum* Groups I and II

LS-BSR [74], a tool for comparing the relatedness of coding sequences among genomes, was used to evaluate the genetic content of Group I and II strains and to cluster strains based upon coding sequence similarity – (Additional file 8: Figure S5). Dendrograms of Group I and II strains constructed by clustering BSR values with an average linkage algorithm (this method considers the entire pan-genome for each Group) show similar overall strain clustering patterns as the core genome phylogenies for both Groups I and II. These clustering patterns indicate that clades identified by core genome phylogenies share coding sequence similarity. Future research could investigate potential functional roles of different clades and could identify marker genes for different clades.

The LS-BSR tool was also applied to investigate the presence or absence of cold shock protein genes in Group II assemblies. Söderholm and colleagues [92] demonstrated that cold shock protein genes, which are commonly found in microbes, were absent from three Group II BoNT/E genome assemblies but were present in the Group II strain Eklund 17B (BoNT/B) genome. Although Group II strains have been shown to grow and produce toxins at low temperatures [93], the LS-BSR approach demonstrated that homologs of cold shock protein genes found in other *C. botulinum* genomes are absent from Group II BoNT/E genomes, while cold shock protein genes are present in the genomes falling into the Group II clade including BoNT/B,/E9 and/F genomes (Eklund 17B, KAPB-3, CDC 66177 and Eklund 202F).

### *bont* gene cluster analyses

The *bont* genes from each of the newly sequenced strains were evaluated to determine if there were any new variants or subtypes. A maximum likelihood phylogenetic tree of *bont* gene sequences extracted from genome assemblies and previously published *bont* gene sequences is presented (Additional file 9: Figure S6). Subtype-specific clades can be seen for serotypes. Recently, new *bont* subtypes have been identified for serotypes A [94] and E [95, 96], and an unusual toxin type (H or F/A) was identified in an isolate from an infant botulism case [4, 5]. However, *bont* gene sequences from newly sequenced strains in this study are closely related (>99 % BLAST identity) to known *bont* subtype gene sequences, indicating that novel *bont* subtypes were not discovered in the newly sequenced strains.

Botulinum neurotoxin genes are associated with two gene cluster types (*ha*+ and *orfX*+) that appear to be located at specific locations in the chromosome or within a plasmid [3]. Information regarding the *bont* type and putative *bont* gene cluster location for draft genome assemblies is included in Table 1. The presence of a marker gene (*PL-6*) for identifying *bont*-containing plasmids [80] in all genome assemblies was determined with BLAST. The *PL-6* gene was present in genome assemblies of published Group I strains known to have plasmids containing *bont* genes (Af84, Bf, CDC 657, CDC 1436, CDC 54075, Loch Maree, Okra, Prevot 594) as well as newly sequenced Group I strains with *bont* genes putatively located on plasmids (10258, AM370, AM553 and AM1195 – all *bont*/B strains). The *PL-6* gene was not identified in assemblies that do not contain *bont*-containing plasmids.

The putative location of toxin gene clusters in the newly sequenced strains was examined and compared to previously published BoNT-producing strain genomes (Table 1). The *bont*/A1 genes in *ha*+ gene clusters have putative chromosomal locations and are associated with *oppA*/*brnQ* operons. The *orfX*+ *bont*/A1 gene in strain U21312 and in serotype A1(B) strains is located within the chromosome and associated with the *arsC* operon, which is also the case in the published *orfX+ bont*/A1 strain CDC 297 [1] and serotype A1(B) strain NCTC 2916 [97]. The *bont*/A2 genes in newly sequenced genomes have putative chromosomal locations and are associated with *orfX+* gene clusters and *arsC* operons, which are common traits for *bont*/A2 genomes [9, 98]. This is in contrast to the bivalent strain CDC 1436 where the *bont*/A2 gene and a *bont*/B5 gene are within a plasmid [1]. Two Argentinian *bont*/A3 strains (U21087 and U21181) and the Loch Maree strain contain *orfX*+ *bont* gene cluster types. The *bont*/A3 gene of the Loch Maree strain is located within a plasmid while the location of the *bont*/A3 gene of the newly sequenced serotype A3 strains is unclear. All of the *bont*/B genes in newly sequenced and published Group I and II strains are putatively found in *ha*+ gene clusters. Interestingly, while the *bont*/B1 gene in the published Okra strain is located on a plasmid [88], the *bont*/B1 gene in newly sequenced strain 20497 has a putative chromosomal location. In contrast to the plasmid location of the *bont*/B2 gene in strain Prevot 594 [1], the *bont*/B2 gene in strain 10159 and strain IBCA1–7060 [4] and *bont*/B3 gene in 20506 have putative chromosomal locations. The *bont*/B6 genes in the newly sequenced Australian strains (AM370, AM553, AM1195) have putative plasmid locations, which is consistent with published *bont*/B6 subtypes from Japan [50]. The *bont*/F genes of newly sequenced Group I strains are found in *orfX+* gene clusters, which is consistent with previously published genomes [9, 98, 99]. All *bont*/F1 genes in strains analyzed in this study are associated with *arsC* operons and have chromosomal locations. Toxin genes in Group I bivalent *bont*/Af strains are associated with *orfX+* gene clusters, and the *bont*/A2 and *bont*/f4 genes have putative chromosomal locations. The *bont*/f5 gene of the trivalent strain Af84 is located within a plasmid [98]. Bivalent *bont*/Ab,/Ba and/Bf strains in Group I have *bont* genes putatively located on plasmids. All the *bont*/E genes in the Group II strains analyzed here are associated with *orfX+* gene clusters and *rarA* operons and have putative chromosomal locations.

## Discussion

Diverse members of the *Clostridia* produce potent botulinum neurotoxins that cause botulism, a flaccid paralysis that affects humans, other mammals, birds and fish. These microbes are of interest for public health and biodefense reasons [100]. Horizontal gene transfer, insertion and recombination events have been documented in BoNT-producing clostridia [1, 7–10], resulting in variation in botulinum neurotoxins, toxin gene clusters and the genomic backgrounds in which the same toxin type or subtype are found. Thus, whole genome sequencing of *Clostridia* strains was conducted to provide insight into toxin gene cluster characterisitics, genomic diversity, phylogenetic relationships and global distribution of BoNT-producing *Clostridia*. Genomic sequence data and draft genome assemblies were generated for 59 *Clostridia* strains from six continents providing a resource to the research community. These data were compared to publicly available whole genome sequence data using multiple methods. Group designation of BoNT-producing strains was achieved with 16S rRNA gene sequence phylogenies and confirmed by phylogenies that included larger swaths of the genome: a phylogeny of concatenated marker genes and a reference-independent, SNP-based phylogeny. While the topologies of the three different phylogenies show some variation, the Group designations of strains are consistent among all three methods. The concatenated marker genes and reference-independent, SNP-based phylogenies demonstrate the diversity within the BoNT-producing Groups I, II and III that has been described with other methods [7, 11, 15, 81, 84].

Core genome phylogenies of Group I and II strains (Figs. 5 and 6) illustrate the phylogenetic diversity within each Group. Homoplasy is distributed throughout the Group I and II core genomes (Fig. 7), which suggests a history of recombination and could affect accurate phylogenetic inference. However, the distinct groupings as well as the diversity of Group I and II strains are apparent. Clustering of genomes based upon gene content (BSR values) produces similar groupings of strains as the core genome phylogenies for both Groups. ANIm values reveal the genomic diversity within both Groups I and II indicating that multiple species, subspecies or genomovars are likely present in each Group [86]. Weigand and colleagues [84] recently delineated Group I *C. botulinum* and *C. sporogenes* strains using similar comparative genomic techniques. The diversity within BoNT-producing *Clostridia* should be considered when studying these microbes. For example, appropriate strains must be chosen for conducting microbiological challenge tests used for evaluating the risk of BoNT-producing *Clostridia* or botulinum neurotoxins contaminating food [101–103].

The core genome phylogenies of Group I and II strains also illustrate the diversity of genomic backgrounds that express the same toxin type or subtype. Examples of different genomic backgrounds containing the same toxin type/subtype are abundant in Group I. Subtype *bont*/A1 strains are found in multiple clades. Argentinian *bont*/A2 strains and *bont*/A2 strain Kyoto-F cluster distantly from the Mauritius *bont*/A2 strain and two other Argentinian *bont*/A2 strains. Two rare *bont*/A3 strains from Argentina cluster distantly from the *bont*/A3 Loch Maree strain from Scotland. A clade of bivalent *bont*/Af strains is distantly related to a clade of bivalent strains containing *bont*/A, *bont*/B and *bont*/F genes indicating the diversity of genomic backgrounds containing different combinations of these three toxin types. Two clades of *bont*/A1(B) strains are distinguished: one clade with isolates from Ecuador, Japan and the US and the other clade with isolates from Japan. Subtype *bont*/B1 and/B2 strains in which the *bont* genes have putative chromosomal locations are not closely related to previously sequenced *bont*/B1 and/B2 strains in which the *bont* genes are located on plasmids [1, 88]. This distribution of toxin types and subtypes throughout the Group I phylogeny suggests a history of horizontal gene transfer.

This study includes BoNT-producing strains isolated from both environmental sources and human botulism cases from across the globe; however, whole genome sequence data for strains from many parts of the world are not available. Large geographical gaps are evident in much of Africa, Asia, and South America suggesting much additional diversity is unsampled and unknown. Diversity of both genomic backgrounds and toxin type is apparent in some geographic regions for which many genome assemblies are available. For example, Argentina is a major reservoir of many serotypes. Interestingly, many BoNT/A2 strains were identified in Argentina, and examination of the Group I core genome phylogeny illustrates the genomic diversity present in this country. While some subtypes have been reported only in certain locales (e.g. *bont*/A2f4 in Argentina [98, 104]), many subtypes and/or strains that are closely related by core genome comparisons are found in distant locations. For instance, BoNT/A1-producing strains are found throughout the world, and *bont*/A1(B) strains showing a close phylogenetic relationship are found in Ecuador, Japan and the United States. Patterns of global serotype and genomic background distribution are not apparent from the analyses presented in this study. Understanding the global distribution of BoNT-producing strains is complex as these *Clostridia* are spore-forming microbes capable of persisting in an environment and can be distributed by a number of dispersal processes. However, a better understanding of the phylogeography of BoNT-producing strains and of the ability for strain attribution in events such as public health emergencies (e.g. foodborne outbreaks) may be possible through additional whole genome sequencing efforts (including producing complete genomes representing the diversity of BoNT-producing strains).

The comparative genomic techniques used in this study are capable of differentiating BoNT-producing *Clostridia* (including strains of the same serotype and from similar geographic locations) and provide a framework for the study of these toxin-producing microbes (e.g. investigating horizontal gene transfer and phylogeography). As more whole genome sequence data for BoNT-producing strains become available our understanding of the diversity and distribution of these microbes has expanded. Recent whole genome sequencing of serotype E strains revealed information on stress response [92] and new *bont*/E toxin variants [95], and whole genome sequencing has identified *bont*/B6 strains in Australia that are closely related to *bont*/B6 strains previously reported in Japan [50]. Future research utilizing the growing number of whole genome sequenced strains will further our understanding of these microbes and potentially aid the development of diagnostics and treatments. For instance, comparative genomics may aid in the identification of marker genes capable of identifying strains within Groups (or specific clades in each Group) more rapidly than methods relying on cultivation and subsequent whole genome sequencing.

## Conclusions

Whole genome sequence analyses of *Clostridia* species illustrate the diversity of botulinum-neurotoxin-producing strains and the plasticity of the genomic backgrounds in which *bont* genes are found. Core genome phylogenies are a powerful tool for differentiating BoNT-producing strains and providing a framework for the study of these bacteria. As more BoNT-producing strains are whole genome sequenced, our understanding of the genomic diversity of microbes capable of producing potent botulinum neurotoxins will continue to expand.

## Availability of supporting data

Whole genome sequence data in support of the results of this article have been deposited in the NCBI Sequence Read Archive (BioProject ID PRJNA286797, study accession number SRP059640). Draft genome assemblies have been deposited in the NCBI WGS database; accession numbers for draft genome assemblies are included in Table 1 and Additional file 1: Table S1. Phylogenetic data is deposited in the TreeBase database (Study 18872) and https://github.com/chawillia/phylogenetic_data_2016.

## Additional files

Additional file 1: Table S1. Information regarding newly sequenced strains analyzed in this study. Additional information regarding newly sequenced strains. (XLSX 22 kb) Additional file 2: Table S2. Information regarding publicly available genomes examined in this study. Table of information regarding genomes included in this study. (XLSX 27 kb) Additional file 3: Table S3. Table of all pairwise ANIm values. Table of pairwise ANIm values computed with JSpecies [72]. (XLSX 168 kb) Additional file 4: Figure S1. Core genome phylogenies of Group I strains. A) Core genome phylogeny of *C. botulinum* Group I strains inferred with RAxML [63] using the ASC_GTRGAMMA model on an alignment of 182,200 core genome SNPs produced with NASP [61] using *C. sporogenes* ATCC 15579 [GenBank:ABKW00000000] as the reference genome. The consistency index is 0.56, and the retention index is 0.91 (computed with the R package phangorn). B) Core genome phylogeny of Group I strains inferred with FastTree2 [54] on a 1780-character core SNP matrix generated with kSNP [59]. The consistency index is 0.64, and the retention index is 0.93. The phylogenies were rooted with the clade that includes *C. sporogenes* and *C. botulinum bont*/B serotypes (bottom of Figure). Gray circles indicate bootstrap values over 95 %. While there are small variations in the phylogenies generated with different methods (Fig. 5 and Additional file 4: Figure S1), the overall topology of the Group I tree appears robust. The pairwise overall topological scores computed by Compare2Trees [69] range from 80 to 86 % for the phylogenies presented in Fig. 5 and (Additional file 4: Figure S1). (PDF 59 kb) Additional file 5: Figure S2. Group I concatenated MLST gene phylogeny. Phylogeny of aligned (MUSCLE [57]) and concatenated MLST genes for Group I genomes inferred with FastTree2 [54]. The MLST profile included *aceK*, *aroE*, *hsp60*, *mdh*, *oppB*, *recA* and *rpoB* [21]. Taxa labeled ST are 83 sequence types available from PubMLST [77, 78]. Investigation of the concatenated MLST gene phylogeny suggests that diverse BoNT-producing strains have yet to be whole genome sequenced. (PDF 31 kb) Additional file 6: Figure S3. Core genome phylogenies of Group II strains. A) Core genome phylogeny of *C. botulinum* Group II strains inferred with RAxML [63] using the ASC_GTRGAMMA model on an alignment of 200,276 core genome SNPs produced with NASP [61] using *C. botulinum* strain Alaska E43 [GenBank:CP001078] as the reference. The consistency index is 0.81, and the retention index is 0.90 (computed with the R package phangorn). B) Core genome phylogeny of Group II *C. botulinum* strains inferred with FastTree2 [54] on a 35,382-character core SNP matrix generated with kSNP [59]. The consistency index is 0.83, and the retention index is 0.90. The phylogenies were rooted with the clade that includes strains Eklund 202F, KAPB-3, Eklund 17B and CDC 66177. Gray circles indicate bootstrap values over 95 %. While there are small variations in the phylogenies generated with different methods (Fig. 6 and Additional file 6: Figure S3), Group II strains are separated into two distinct clades. The pairwise overall topological scores computed by Compare2Trees [69] range from 93 to 100 %. (PDF 35 kb) Additional file 7: Figure S4. Group II concatenated MLST gene phylogeny. Phylogeny of aligned (MUSCLE [57]) and concatenated MLST genes for Group II genomes inferred with FastTree2 [54]. The MLST profile included 16S rRNA genes, *atpD*, *guaA*, *gyrB*, *ilvD*, *lepA*, *mutL*, *oppB*, *pta*, *pyc*, *recA*, *rpoB*, *trpB* and *tuf* [22]. Taxa labeled *C. botulinum* are WGS samples. Taxa labeled with single-word name include serotype E strains for which the MLST genes were sequenced by MacDonald and colleagues [22]. (PDF 37 kb) Additional file 8: Figure S5. Dendrograms clustering Group I and Group II strains by BSR values. A) A dendrogram generated by clustering Group I strains by BSR values with an average linkage method in MeV [76]. B) A dendrogram generated by clustering Group II strains by BSR values with an average linkage method in MeV [76]. (PDF 41 kb) Additional file 9: Figure S6.
*bont* gene phylogeny. A phylogeny inferred with FastTree2 [54] on a nucleotide alignment (MUSCLE [57]) of botulinum neurotoxin genes. Gray circles indicate bootstrap values over 90 %. The tree was rooted with tetanus toxin gene sequences in FigTree [55]. (PDF 54 kb)

## Abbreviations

AFLP: amplified fragment length polymorphism
ANIm: average nucleotide identity (MUMmer method)
BLAST: basic local alignment search tool
BoNT: botulinum neurotoxin
*bont*: botulinum neurotoxin gene
*C. botulinum*: *Clostridium botulinum*
DNA: deoxyribonucleic acid
IS: insertion sequence
LS-BSR: large-scale BLAST score ratio
MeV: MultiExperiment viewer
MLST: multi-locus sequence typing
NCBI: National Center for Biotechnology Information
PCR: polymerase chain reaction
PFGE: pulsed-field gel electrophoresis
rRNA: ribosomal ribonucleic acid
SNP: single nucleotide polymorphism
ST: sequence type
WGS: whole genome sequence
