# Supplementary figures and images for: Comparative genomic analyses reveal broad diversity in botulinum-toxin-producing Clostridia
Source: BMC Genomics. 2016 Mar 3;17:180. doi: 10.1186/s12864-016-2502-z (PMC4778365; doi:10.1186/s12864-016-2502-z)

A

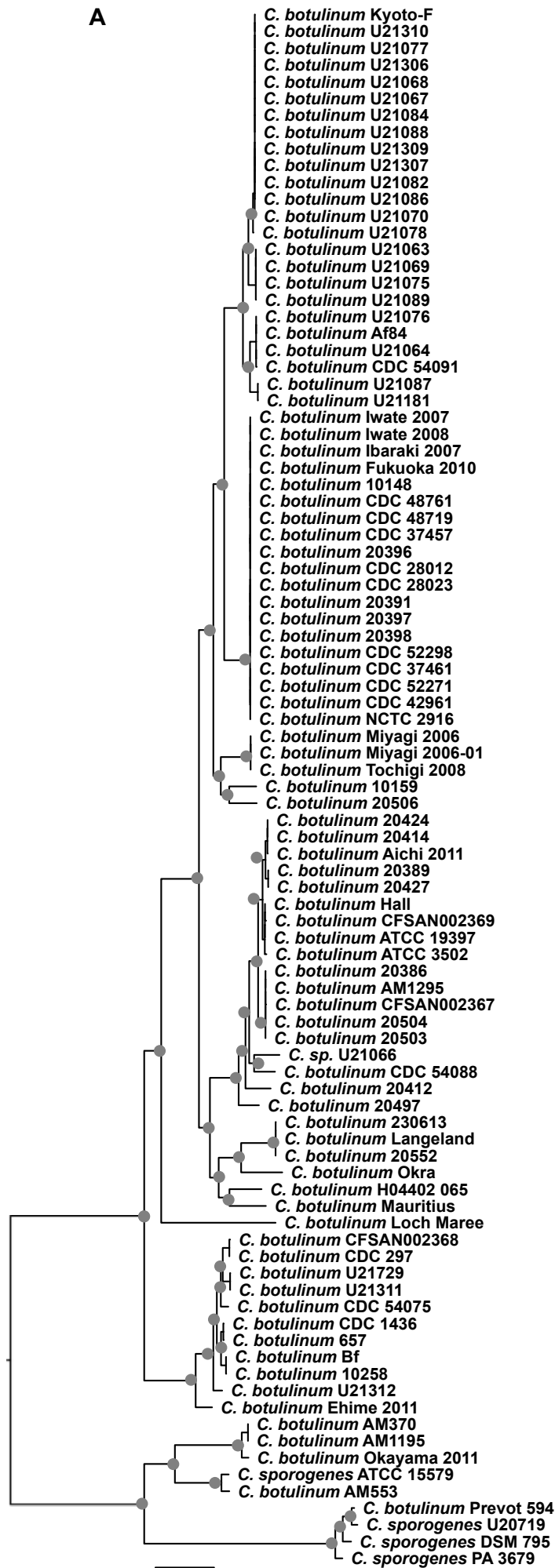

0.05

B

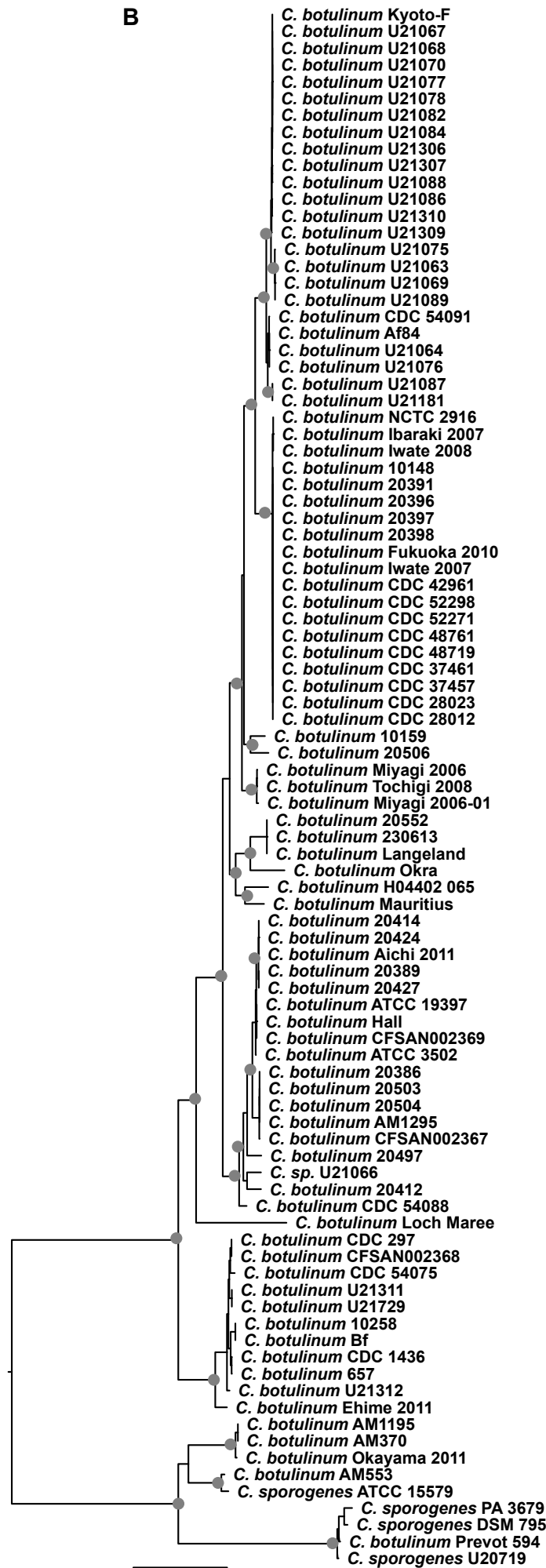

0.2

Supplement: Additional file 4: Figure S1. — Core genome phylogenies of Group I strains. A) Core genome phylogeny of C. botulinum Group I strains inferred with RAxML [63] using the ASC_GTRGAMMA model on an alignment of 182,200 core genome SNPs produced with NASP [61] using C. sporogenes ATCC 15579 [GenBank:ABKW00000000] as the reference genome. The consistency index is 0.56, and the retention index is 0.91 (computed with the R package phangorn). B) Core genome phylogeny of Group I strains inferred with FastTree2 [54] on a 1780-character core SNP matrix generated with kSNP [59]. The consistency index is 0.64, and the retention index is 0.93. The phylogenies were rooted with the clade that includes C. sporogenes and C. botulinum bont/B serotypes (bottom of Figure). Gray circles indicate bootstrap values over 95 %. While there are small variations in the phylogenies generated with different methods (Fig. 5 and Additional file 4: Figure S1), the overall topology of the Group I tree appears robust. The pairwise overall topological scores computed by Compare2Trees [69] range from 80 to 86 % for the phylogenies presented in Fig. 5 and (Additional file 4: Figure S1). (PDF 59 kb) [file 12864_2016_2502_MOESM4_ESM.pdf]

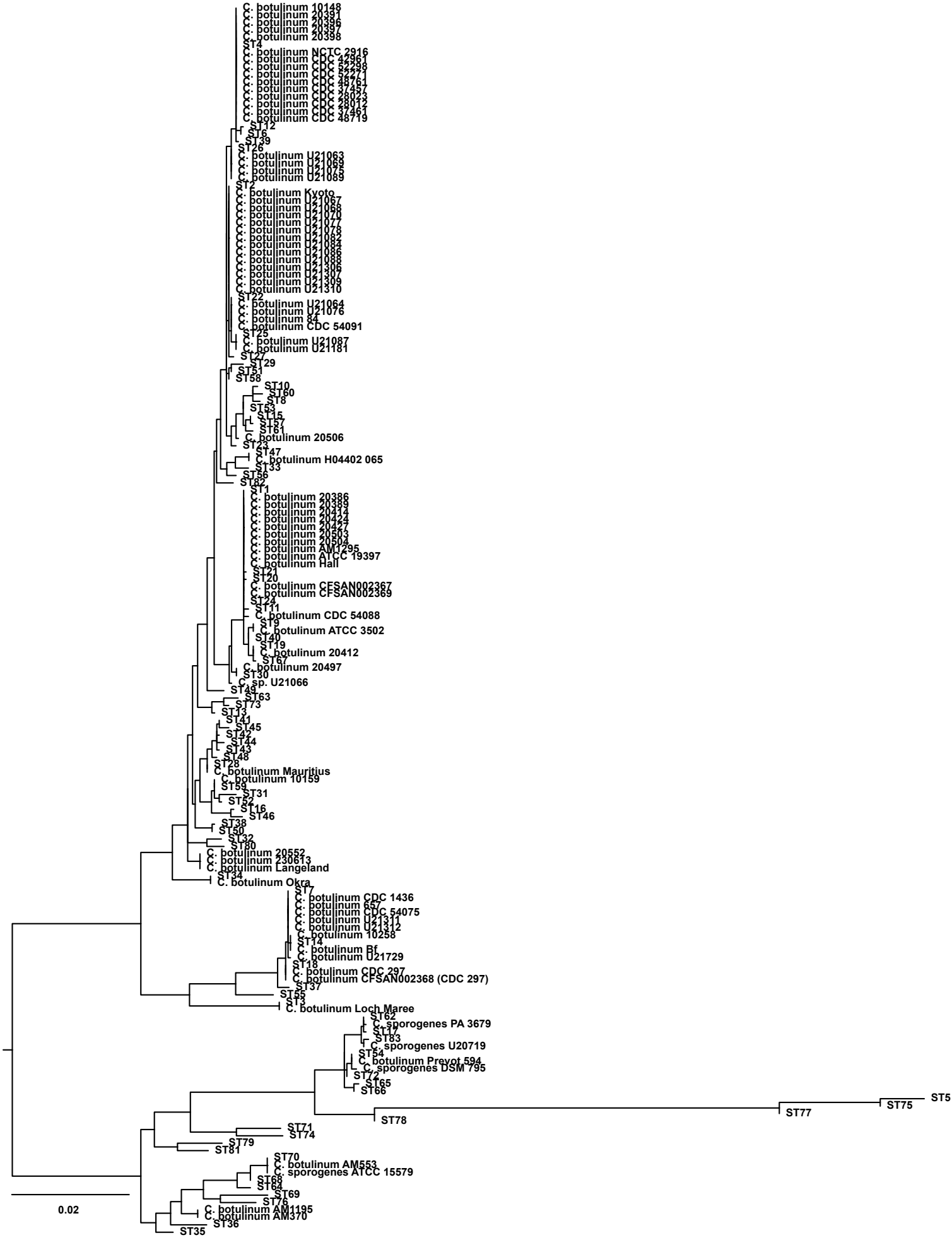

Supplement: Additional file 5: Figure S2. — Group I concatenated MLST gene phylogeny. Phylogeny of aligned (MUSCLE [57]) and concatenated MLST genes for Group I genomes inferred with FastTree2 [54]. The MLST profile included aceK, aroE, hsp60, mdh, oppB, recA and rpoB [21]. Taxa labeled ST are 83 sequence types available from PubMLST [77, 78]. Investigation of the concatenated MLST gene phylogeny suggests that diverse BoNT-producing strains have yet to be whole genome sequenced. (PDF 31 kb) [file 12864_2016_2502_MOESM5_ESM.pdf]

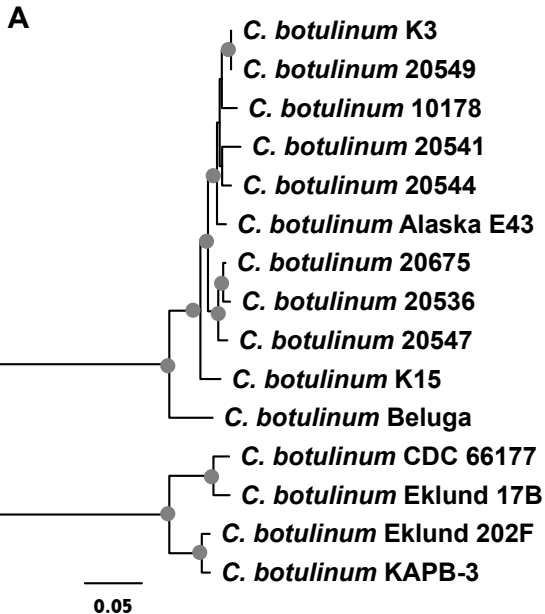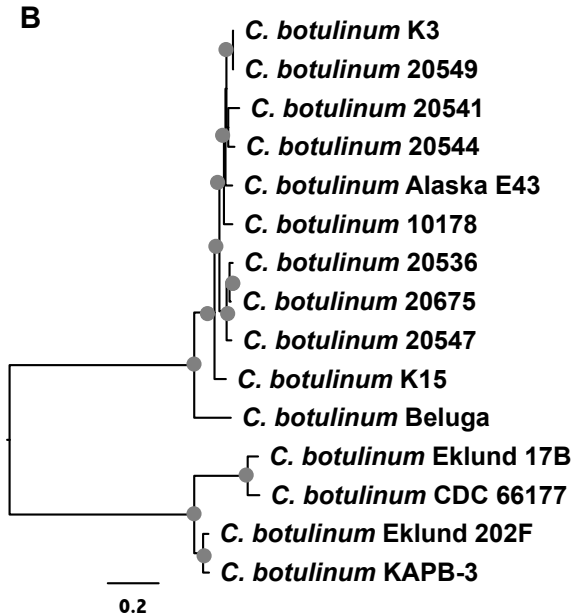

Supplement: Additional file 6: Figure S3. — Core genome phylogenies of Group II strains. A) Core genome phylogeny of C. botulinum Group II strains inferred with RAxML [63] using the ASC_GTRGAMMA model on an alignment of 200,276 core genome SNPs produced with NASP [61] using C. botulinum strain Alaska E43 [GenBank:CP001078] as the reference. The consistency index is 0.81, and the retention index is 0.90 (computed with the R package phangorn). B) Core genome phylogeny of Group II C. botulinum strains inferred with FastTree2 [54] on a 35,382-character core SNP matrix generated with kSNP [59]. The consistency index is 0.83, and the retention index is 0.90. The phylogenies were rooted with the clade that includes strains Eklund 202F, KAPB-3, Eklund 17B and CDC 66177. Gray circles indicate bootstrap values over 95 %. While there are small variations in the phylogenies generated with different methods (Fig. 6 and Additional file 6: Figure S3), Group II strains are separated into two distinct clades. The pairwise overall topological scores computed by Compare2Trees [69] range from 93 to 100 %. (PDF 35 kb) [file 12864_2016_2502_MOESM6_ESM.pdf]

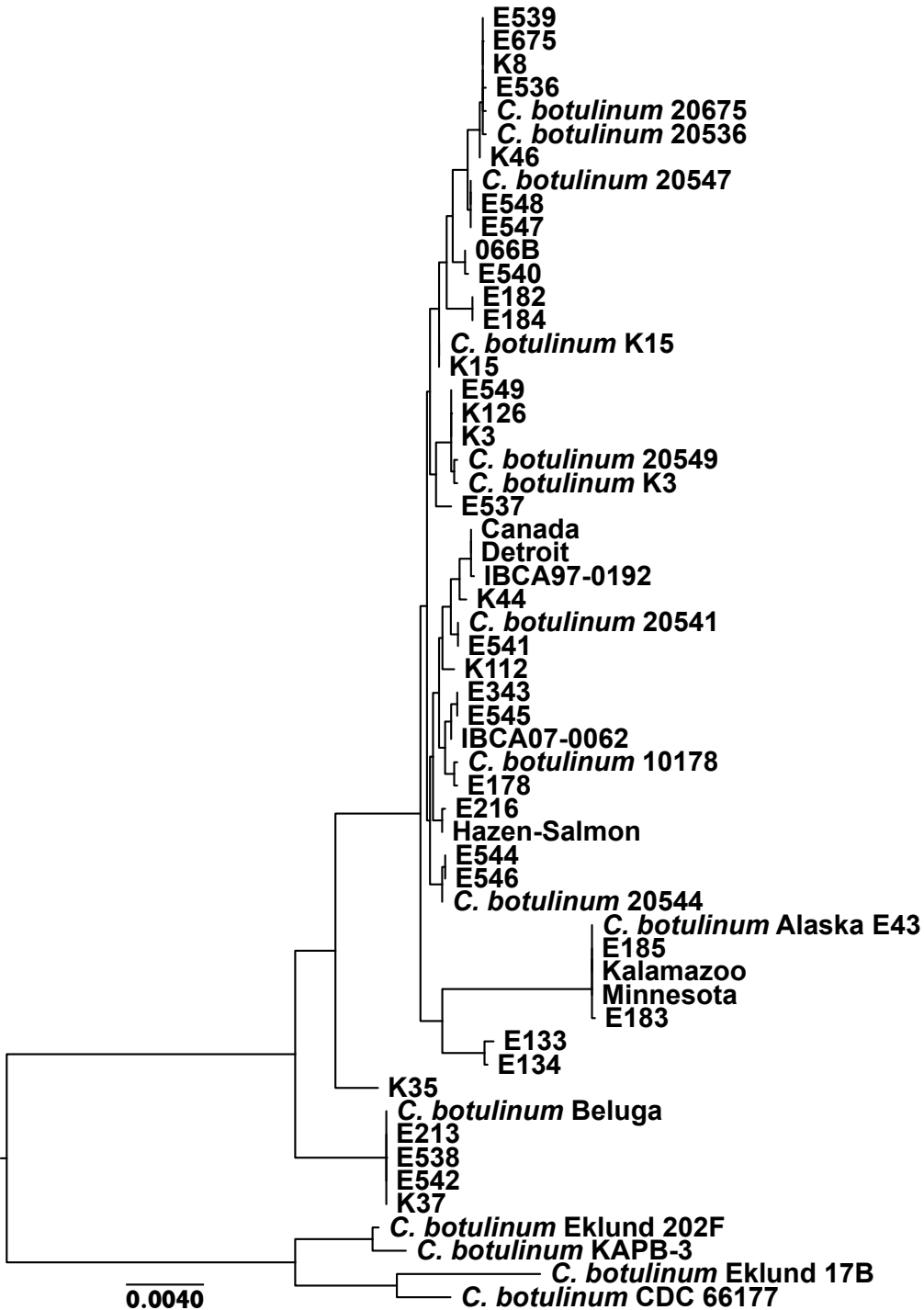

Supplement: Additional file 7: Figure S4. — Group II concatenated MLST gene phylogeny. Phylogeny of aligned (MUSCLE [57]) and concatenated MLST genes for Group II genomes inferred with FastTree2 [54]. The MLST profile included 16S rRNA genes, atpD, guaA, gyrB, ilvD, lepA, mutL, oppB, pta, pyc, recA, rpoB, trpB and tuf [22]. Taxa labeled C. botulinum are WGS samples. Taxa labeled with single-word name include serotype E strains for which the MLST genes were sequenced by MacDonald and colleagues [22]. (PDF 37 kb) [file 12864_2016_2502_MOESM7_ESM.pdf]

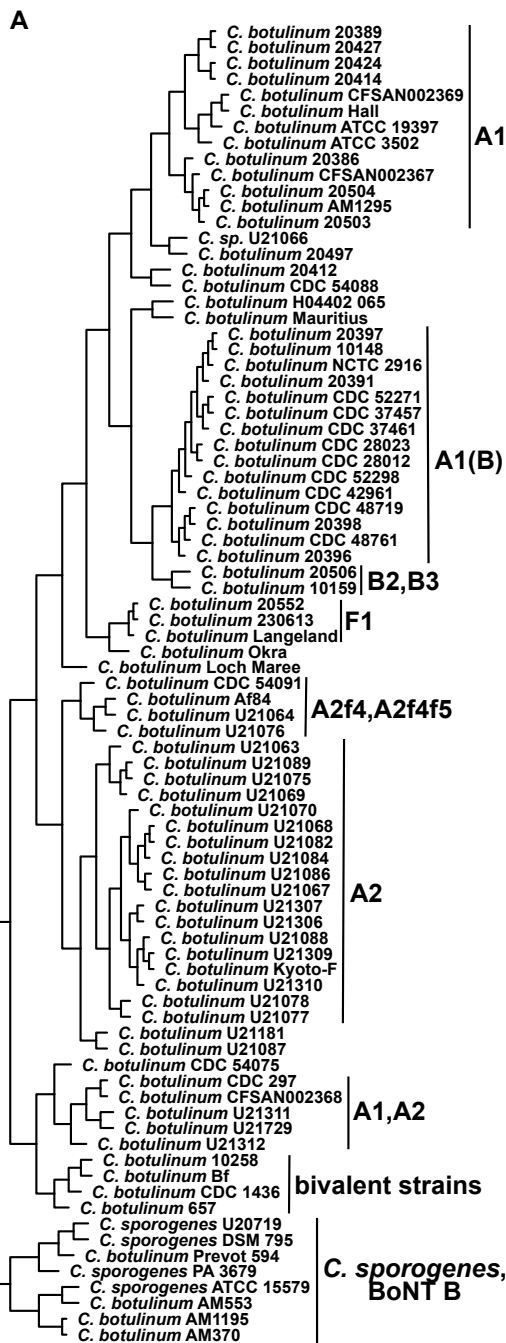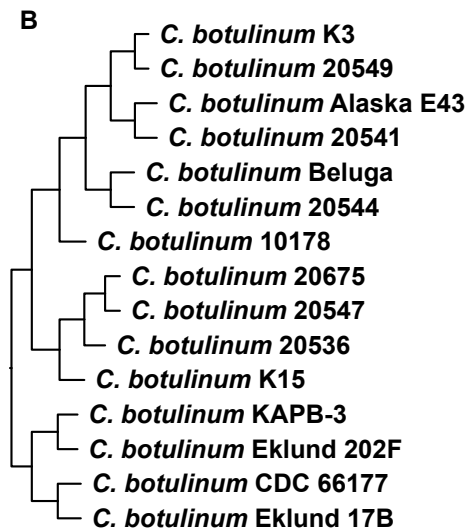

Supplement: Additional file 8: Figure S5. — Dendrograms clustering Group I and Group II strains by BSR values. A) A dendrogram generated by clustering Group I strains by BSR values with an average linkage method in MeV [76]. B) A dendrogram generated by clustering Group II strains by BSR values with an average linkage method in MeV [76]. (PDF 41 kb) [file 12864_2016_2502_MOESM8_ESM.pdf]

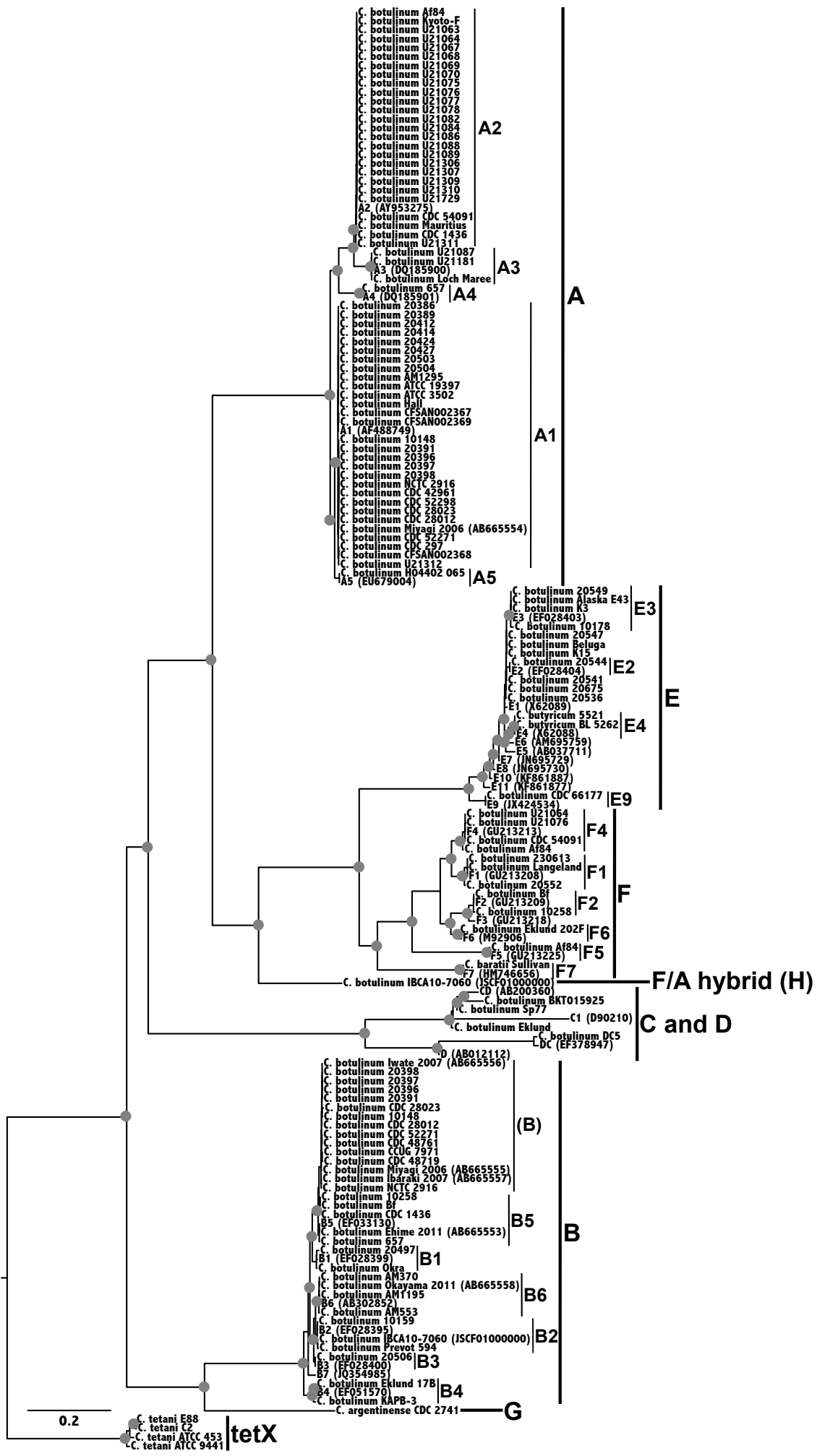

Supplement: Additional file 9: Figure S6. — bont gene phylogeny. A phylogeny inferred with FastTree2 [54] on a nucleotide alignment (MUSCLE [57]) of botulinum neurotoxin genes. Gray circles indicate bootstrap values over 90 %. The tree was rooted with tetanus toxin gene sequences in FigTree [55]. (PDF 54 kb) [file 12864_2016_2502_MOESM9_ESM.pdf]
